# O-GlcNAc modification is associated with insulin sensitivity in the whole blood of healthy young adult males

**DOI:** 10.1186/1758-5996-6-96

**Published:** 2014-09-09

**Authors:** Jason P Myslicki, Jane Shearer, Dustin S Hittel, Curtis C Hughey, Darrell D Belke

**Affiliations:** Faculty of Kinesiology, University of Calgary, 3300 University Drive NW, Calgary, Alberta T2N 4N1 Canada; Department of Biochemistry and Molecular Biology, Faculty of Medicine, University of Calgary, Calgary, Alberta T2N 1N4 Canada

**Keywords:** Young adult, Metabolism, HOMA-IR, Obesity, Insulin resistance

## Abstract

**Background:**

Hemoglobin A1c (HbA1c) is the predominant diagnostic tool for diabetes diagnosis and progression. However, it has proven to be insensitive at pre-diabetic threshold values. O-linked-*β*-*N*-acetylglucosamine (O-GlcNAc) modification has emerged as a sensitive biomarker. The purpose of this study was to explore the sensitivity of O-GlcNAc expression as a potential marker of early metabolic dysfunction in a young adult population. Healthy, young males (18–35 y) from the *A*ssessing *I*nherited *M*etabolic syndrome *M*arkers in the *Y*oung study (*AIMMY*), were divided into low (LH,0.60) or high (HH,1.61) homeostatic model assessment of insulin resistance (HOMA-IR) cohorts.

**Findings:**

The relationships between a panel of anthropometric, metabolic measures and whole blood global protein O-GlcNAc was examined. O-GlcNAc and O-GlcNAc transferase (OGT) levels were quantified by immunoblotting and compared to anthropometric measures: body mass index (BMI), percentage body fat, aerobic fitness, blood glucose, triglycerides, HDL, insulin, and HbA1c. HOMA-IR cohorts showed no differences in BMI, blood glucose or HbA1c, but differed in percent body fat, plasma triglycerides, and circulating insulin. Greater O-GlcNAc expression was observed in the whole blood of HH compared to LH. Moreover, a positive association between HOMA-IR and O-GlcNAc emerged, while no relationship was found between HbA1c and HOMA-IR. This effect was not related to OGT expression.

**Conclusions:**

Results indicate that O-GlcNAc has a greater sensitivity to metabolic status compared to HbA1c in this population. O-GlcNAc has the potential to serve as a screening tool for predicting future metabolic disturbances in a young healthy adult population free of any clinically relevant pathologies.

**Electronic supplementary material:**

The online version of this article (doi:10.1186/1758-5996-6-96) contains supplementary material, which is available to authorized users.

## Introduction

Current estimates indicate there are 347 million diagnosed cases of diabetes worldwide, 90% of these being attributable to type 2 diabetes (T2D) [1]. Research establishes that metabolic diseases, including T2D are often initiated in childhood. In fact, the extent of metabolic disease risk in children and young adults is correlated with the presence of the same risk factors identified in adults including obesity and insulin resistance [2].

At present, haemoglobin A1c (HbA1c) is the preferred biomarker for diabetes diagnosis and monitoring; however, this marker has proven to be insensitive at pre-diabetic and diabetic threshold values [3]. As a result, there has been an increased prevalence of undiagnosed diabetes, thereby increasing the potential for complications. Recently, the post-translational protein modification, O-linked *β*-*N*-acetylglucosamine (O-GlcNAc), has emerged as a potential biomarker for T2D. Protein O-GlcNAc is the post-translational modification of intracellular proteins by the addition or removal of O-GlcNAc mediated by β-N-acetylglucosaminyltransferase (OGT) and β-N-acetylglucosaminidase (OGA) in a manner analogous to phosphorylation [4]. As the O-GlcNAc pathway utilizes uridine diphosphate N-acetylglucosamine from the hexosamine biosynthetic pathway, its study has been closely linked to circulating glucose levels and diabetes, where it is thought to play a role in mediating insulin resistance [5, 6].

Aberrant protein O-GlcNAc modification has been associated with hyperglycemia, insulin resistance and metabolic dysfunction [4, 7]. To date, studies have examined changes in the O-GlcNAc of erythrocytes and leukocytes of healthy, pre-diabetic and overtly diabetic adults in order to gain an enhanced understanding of this modification as it relates to the development of the disease [4, 7, 8]. These studies show that in overt diabetes, O-GlcNAc analysis has the potential to identify metabolic disturbances better than HbA1c [7, 8]. However, nothing is known about O-GlcNAc levels in a clinically healthy young, asympotmatic adult population. Study aims were to explore the relationships between a panel of anthropometric and metabolic parameters with whole blood O-GlcNAc, OGT and OGA in a sample of healthy young adults.

## Methods

Whole blood samples (n = 24) were collected from the Assessing Inherited Markers of Metabolic Syndrome in the Young (AIMMY) cohort at the University of Calgary and approved by the Conjoint Health Research Ethics Board (ID: E23521). Individual subjects were chosen from the entire AIMMY cohort (n = 122) based on their Homeostatic Model of Assessment – Insulin Resistance (HOMA-IR) classification, being in either below lowest (LL, P25, <0.845) or greatest (HH, P75, >1.61) quartiles for this measure. HOMA-IR calculated as: [HOMA-IR = [Fasting glucose (mmol · L^−1^) × fasting insulin (pmol)]/22.5]. The inclusion criteria were 1) age 18–35 years, 2) post-pubescent and 3) willing and able to provide informed consent. All subjects were considered healthy as previously outlined [9]. The study required subjects to fast for 12 h prior to blood collection [9]. Body mass index (BMI), percentage body fat (%), blood measures including glucose, triglycerides, high density lipoprotein cholesterol, insulin, and HbA1c were collected and analyzed as described [9].

Whole blood samples were collected and immediately frozen. In preparation for immunoblotting, blood samples were homogenized in a lysis buffer (pH 7.4) consisting of 20 mM NaCl, 20 mM Tris–HCl, 0.1 mM EDTA, 1% Triton X-100, 0.5% (wt./vol.) sodium deoxycholate, and 0.1% β-mercaptoethanol (vol./vol.) in the presence of phosphatase inhibitor, protease inhibitor, and PUGNAc (N-acetylhexosaminidase inhibitor, Sigma-Aldrich, St. Louis, MO, A7229) to inhibit O-GlcNAcase activity. Blood proteins were detected as previously described [10, 11]. Primary antibodies included anti-RL2 (1:1000; Abcam, ab2739), anti-OGT (1:1000; DM-17 Sigma-Aldrich, St. Louis, MO), and anti-OGA (1:1000; Santa Cruz Biotech, sc-135093) with glyceraldehyde 3-phosphate dehydrogenase (anti-GAPDH) (1:1000; Abcam, ab9485) serving as a control. Secondary antibodies were as follows: anti-RL2: goat anti-mouse IgG HRP conj. (1:2000, Thermo Scientific, 32430); and anti-OGT, anti-OGA, and anti-GAPDH: goat anti-rabbit IgG HRP (1:2000, Cell Signaling Technology, 7074). Signals were detected employing chemiluminesce (West Femto, Thermo Fisher Scientific) and then exposed and analyzed using Syngene ChemiGenius^2^ Bio Imaging System.

**Figure 1:**
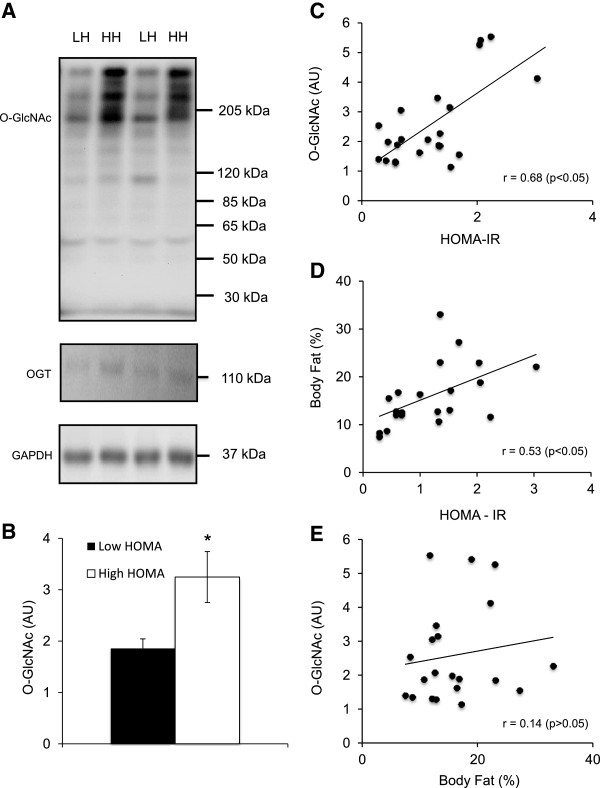
**Analysis of Blood for O-GlcNAc levels in Relation to Anthropometric Measurements. A)** Analysis of whole blood comparing subjects with low (LH) and high (HH) homeostatic model assessment for insulin resistance (HOMA-IR) for global O-GlcNAcylation (O-GlcNAc), β-N-acetylglucosaminyltransferase (OGT), and glyceraldehyde 3-phosphate dehydrogenase (GAPDH) in arbitrary units (AU). **B)** Quantification of global O-GlcNAc for LH and HH. **C)** Linear regression analysis of O-GlcNAc and HOMA-IR. **D)** Linear regression analysis of body fat percentage (%) and HOMA-IR. **E)** Linear regression analysis of O-GlcNAc and body fat percentage (%). All data represents n = 24. *Indicates p < 0.05.

Subject characteristics and metabolic markers (Table 1, Figure 1B) were compared employing independent t-tests at a α = 0.05 significance level (SPSS Statistics V20). The Pearson Product–moment Correlation Coefficient was determined and compared to a critical value for a two-tailed test (Figures 1C-E) [12]. All data are reported as mean ± standard error of measurement (SEM).

**Table 1:** **Baseline characteristics and metabolic markers of all subjects and stratified by low and high homeostatic model assessment, insulin resistance (HOMA-IR) scores**

	All Subjects (n = 24)	Low HOMA-IR (n = 12)	High HOMA-IR (n = 12)
Age (years)	24 ± 1	24 ± 1	24 ± 1
Mean HOMA–IR	1.2 ± 0.2	0.5 ± 0.1	1.9 ± 0.2
BMI	24.7 ± 0.6	23.9 ± 0.8	25.5 ± 0.7
Body Fat (%)	16.3 ± 1.4	12.2 ± 0.9	20.3 ± 2.2*
Glucose (mM)	4.5 ± 0.1	4.6 ± 0.1	4.5 ± 0.1
Triglycerides (mM)	1.0 ± 0.1	0.8 ± 0.1	1.2 ± 0.1*
HDL Cholesterol (mM)	1.5 ± 0.1	1.7 ± 0.1	1.4 ± 0.1
Insulin (pM)	43.4 ± 6.0	19.8 ± 2.3	65.1 ± 6.6*
HbA1c (%)	5.5 ± 0.1	5.5 + 0.1	5.5 + 0.1
VO_2 peak_ (20s) (ml⋅kg^−1^⋅min^−1^)	49.1 ± 1.8	52.2 ± 2.4	45.7 ± 2.5

Abbreviations are as follows: body mass index (BMI, kg⋅m^−2^), high-density lipoprotein cholesterol (HDL, mM), glycated hemoglobin (HbA1c, %), aerobic capacity (VO_2 peak,_ ml⋅kg^−1^⋅min^−1^). Values are means ± SEM. *Indicates there is a significant difference (p < 0.05) between low and high HOMA-IR groups.

## Results

Subject characteristics are presented in Table 1. HOMA–IR for the LH and HH group were 0.5 ± 0.1 and 1.9 ± 0.2 respectively. These values were much lower than the widely adopted HOMA-IR cut-off of 2.60 for insulin resistance [13]. No individuals were obese (BMI >30) or pre-diabetic. The current diabetes diagnostic method, HbA1c, was unable to distinguish the two groups, LH and HH, with respect to insulin resistance (p > 0.05).

Global O-GlcNAc modification was greater in HH compared to LH (p = 0.02) (Figure 1A, B). Results show that this effect was not related to a difference in OGT expression (Figure 1A) and we were unable to attain any consistent OGA signal with immunoblotting, hence we did not include these data. OGA has been shown to be vulnerable to freeze thaw cycles [7, 14], possibly explaining why no signal was detected. All correlations are exhibited in Figure 1C including the noteworthy positive correlation between O-GlcNAc and HOMA-IR (r = 0.68, p < 0.05) (Figure 1C). Traditional metabolic disease markers, such as BMI, body fat percentage, plasma HDL and triglycerides all had moderate to strong relationships with HOMA-IR. The relationship between body fat percentage and HOMA-IR is shown in Figure 1D (r = 0.53, p < 0.05). The relationship between O-GlcNAc and body fat percentage is shown in Figure 1E (r = 0.14, p > 0.05). There was no statistically significant difference in VO_2_ peak (20s) between LH and HH (p = 0.07), nor a relationship between O-GlcNAc and VO_2_ (Additional file 1: Table S1).

## Discussion and conclusion

Many metabolic disease risk factors, including insulin resistance begin to accumulate in young adulthood [15]. Consequently, there is an acute need to establish a sensitive biomarker of diabetes prior to initiation of the disease in this population. This study explored the relationship between whole protein O-GlcNAc and HOMA-IR in a healthy young adult population, free of any clinically relevant pathology involving metabolism. O-GlcNAc may be an ideal marker because intracellular O-GlcNAc modification of proteins serves as a cellular tool for nutrient sensing [16] and cellular protection [17]. In metabolic disease states such as type 2 diabetes, hyperglycemia drives O-GlcNAc and the chronic modification of selected proteins including those involved in insulin signalling [18]. The chronic modification of certain proteins has also been linked to T2D aetiology and progression [4, 5, 16].

Results of the present study show that O-GlcNAc modification can be used to discriminate between LH and HH in a young, healthy population. In contrast, no differences were observed in HbA1c, the current diabetes diagnostic gold standard. This suggests that that O-GlcNAc analysis exhibits greater sensitivity and could potentially serve as a better screening tool for identifying the risk for future metabolic disturbances in a non-overtly diabetic population. These results concur with those of Springhorn et al. [8] who demonstrated that leukocytes from both pre-diabetic and diabetic individuals have elevated global O-GlcNAc compared to healthy controls. The positive correlation between O-GlcNAc and HOMA-IR suggests that the two indices will continue to rise simultaneously but, due to unique personal baseline measurements, determining a threshold cut-off for identifying a given pathology is unrealistic. As such, O-GlcNAc modification may be better utilized as a screening tool and for individual monitoring.

The lack of difference in OGT expression suggests that changes in O-GlcNAc modification are likely due to other enzymatic activity, such as decreased OGA in the HH group, or simply elevated circulating glucose levels and subsequently increased flux through the hexosamine biosynthetic pathway, which is associated with insulin resistance. Biochemically, altered glucose metabolism is a requisite for aberrant O-GlcNAc expression and, in general, nutritional intake, whether it is fatty acid, nucleotide, or glucose, has demonstrated an effect on O-GlcNAcylation as UDP-GlcNAc, the donor molecular for O-GlcNAc, sits at the nexus of these metabolic pathways [19]. As such, lifestyle factors such as diet and exercise should be expected to play a key role in O-GlcNAc modification. In the subjects examined, O-GlcNAc modification did not correlate with VO_2 max_ suggesting that O-GlcNAc modification does not provide insight into an individual’s aerobic capacity or pulmonary functional competence. To date, relatively little is known regarding the effect of chronic exercise on O-GlcNAc modification in blood to help explain this relationship.

Given the lack of studies examining differences in protein O-GlcNAc in a young clinically healthy population, especially with respect to susceptibility towards chronic disease development later in life, our observations provide a foundation for long-term studies of O-GlcNAc as a sensitive screening tool for risk of future metabolic disturbances. We acknowledge the practical limitations associated with administering protein O-GlcNAc quantification via immunoblot analysis to large populations, nonetheless this preliminary research demonstrates the utility of O-GlcNAc analysis as a screening tool. Moving forward, prospective studies following O-GlcNAc modifications over time along with changes in health status, insulin resistance, or the progression of diagnosed diabetes are warranted.

## Electronic supplementary material

Additional file 1: Table S1: Correlation table illustrating r values for the parameters assessed in this study. Abbreviations: percentage body fat (BF %); body mass index (BMI, kg⋅m-2); GlcNAcylation (O-GlcNAc, AU); glycated hemoglobin A1c (HbA1c, %); high density lipoproteins (HDL, mM); homeostatic model assessment for insulin resistance score (HOMA-IR, AU); aerobic capacity (VO2 peak, ml⋅kg-1⋅min-1) and triglycerides (TG, mM). The correlation coefficients were determined using Microsoft Excel:Mac 2011 (Version 14.4.2) The critical value of the Pearson Product-Moment Correlation Coefficient for a two tailed test at a significance level of 0.05 is >0.43 (Galton, 1888). (PPT 114 KB)
