# Supplementary material for: O-GlcNAc modification is associated with insulin sensitivity in the whole blood of healthy young adult males
Source: Diabetol Metab Syndr. 2014 Sep 9;6:96. doi: 10.1186/1758-5996-6-96 (PMC4164748; doi:10.1186/1758-5996-6-96)
Supplement: Supplementary file 1 — Additional file 1: Table S1: Correlation table illustrating r values for the parameters assessed in this study. Abbreviations: percentage body fat (BF %); body mass index (BMI, kg⋅m-2); GlcNAcylation (O-GlcNAc, AU); glycated hemoglobin A1c (HbA1c, %); high density lipoproteins (HDL, mM); homeostatic model assessment for insulin resistance score (HOMA-IR, AU); aerobic capacity (VO2 peak, ml⋅kg-1⋅min-1) and triglycerides (TG, mM). The correlation coefficients were determined using Microsoft Excel:Mac 2011 (Version 14.4.2) The critical value of the Pearson Product-Moment Correlation Coefficient for a two tailed test at a significance level of 0.05 is >0.43 (Galton, 1888). (PPT 114 KB) [file 13098_2014_357_MOESM1_ESM.ppt]

## Slide 1
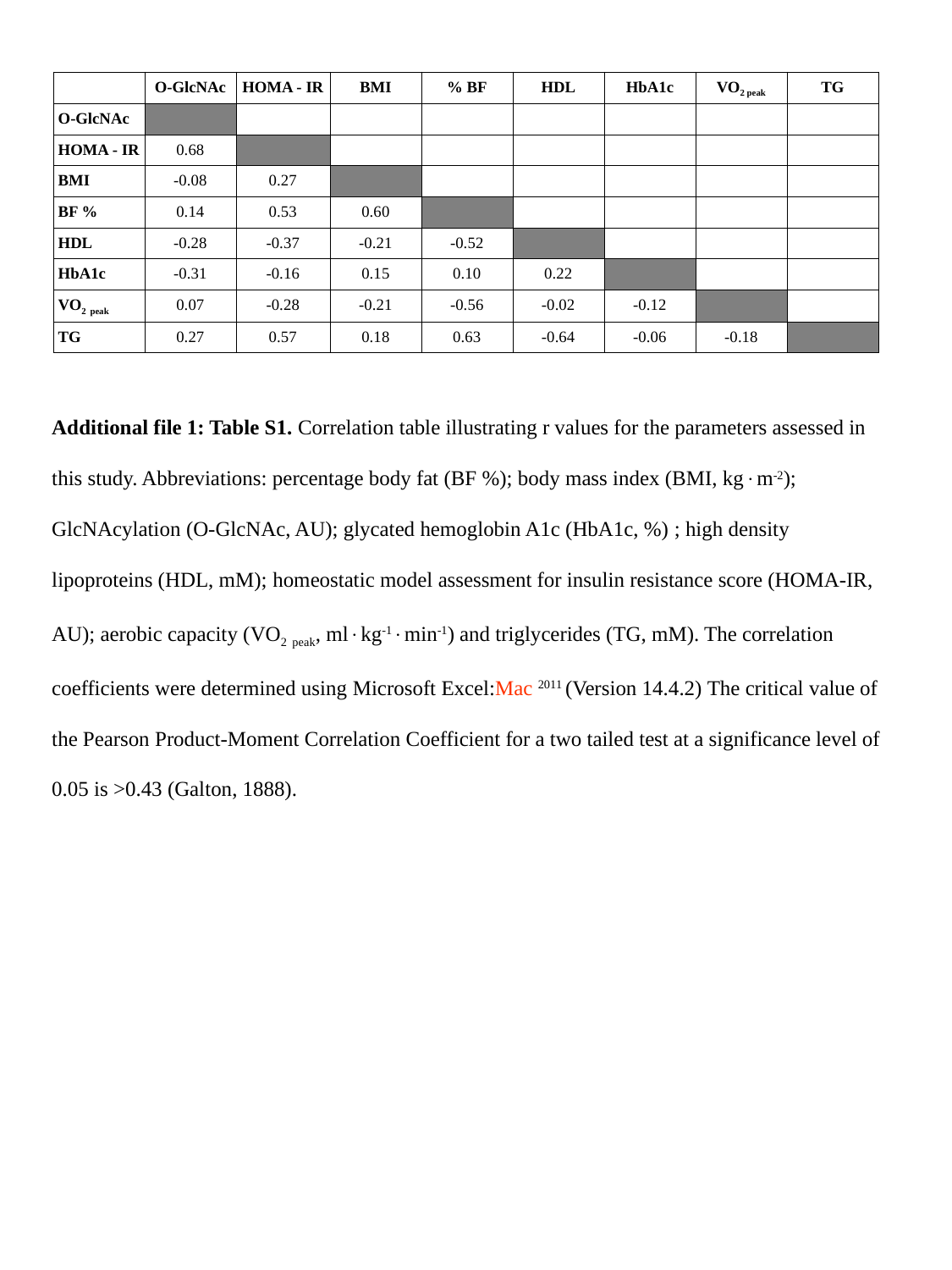

| | O-GlcNAc | HOMA - IR | BMI | % BF | HDL | HbA1c | VO2 peak | TG |
| --- | --- | --- | --- | --- | --- | --- | --- | --- |
| O-GlcNAc | | | | | | | | |
| HOMA - IR | 0.68 | | | | | | | |
| BMI | -0.08 | 0.27 | | | | | | |
| BF % | 0.14 | 0.53 | 0.60 | | | | | |
| HDL | -0.28 | -0.37 | -0.21 | -0.52 | | | | |
| HbA1c | -0.31 | -0.16 | 0.15 | 0.10 | 0.22 | | | |
| VO2 peak | 0.07 | -0.28 | -0.21 | -0.56 | -0.02 | -0.12 | | |
| TG | 0.27 | 0.57 | 0.18 | 0.63 | -0.64 | -0.06 | -0.18 | |
Additional file 1: Table S1. Correlation table illustrating r values for the parameters assessed in this study. Abbreviations: percentage body fat (BF %); body mass index (BMI, kgm-2); GlcNAcylation (O-GlcNAc, AU); glycated hemoglobin A1c (HbA1c, %) ; high density lipoproteins (HDL, mM); homeostatic model assessment for insulin resistance score (HOMA-IR, AU); aerobic capacity (VO2 peak, mlkg-1min-1) and triglycerides (TG, mM). The correlation coefficients were determined using Microsoft Excel:Mac 2011 (Version 14.4.2) The critical value of the Pearson Product-Moment Correlation Coefficient for a two tailed test at a significance level of 0.05 is >0.43 (Galton, 1888).
